# The Influence of Undergraduate Entrepreneurship Education on Entrepreneurial Intention: Evidence From Universities in China’s Pearl River Delta

**DOI:** 10.3389/fpsyg.2021.732659

**Published:** 2021-12-03

**Authors:** Jing Li, Shi-zheng Huang, Ka Yin Chau, Liqiong Yu

**Affiliations:** ^1^School of Business, Macau University of Science and Technology, Macao, Macao SAR, China; ^2^School of Economics and Management, Guangdong University of Petrochemical Technology, Maoming, China; ^3^Faculty of Business, City University of Macau, Macao, Macao SAR, China; ^4^Faculty of International Tourism and Management, City University of Macau, Macao, Macao SAR, China

**Keywords:** entrepreneurship education, entrepreneurial strategy, exploitation innovation, government subsidies, entrepreneurial intention

## Abstract

Entrepreneurship may be taught, and entrepreneurship education is flourishing at colleges and universities. However, previous documents show that entrepreneurship education is inconsistent with the research conclusions of entrepreneurial intention, which is a lack of discussion on the mediating effect of government subsidies from external resources. Based on the cognitive behavior theory, a mediating effect of entrepreneurship education on entrepreneurial strategy and entrepreneurial intention is built. By collecting the data of 334 questionnaires of college students in Pearl River Delta in China, a structural equation is used for empirical analysis. The result indicates that entrepreneurship education does not have a significant influence on entrepreneurial intention; exploration innovation and exploitation innovation have a positive influence on entrepreneurial intention, and exploration innovation and exploitation innovation have a mediating effect on entrepreneurship education and entrepreneurial intention; government subsidies have a positive regulating effect on exploration innovation, exploitation innovation, and entrepreneurial intention. In this article, the application of the cognitive behavior theory in the field of entrepreneurship research is expanded to provide the theoretical basis for building the entrepreneurship education ecosystem, which is conductive to innovation and entrepreneurship to promote regional economic development.

## Introduction

Since Harvard Business School opened the first entrepreneurship course in 1947, entrepreneurship education has risen in the world and attracted widespread attention from entrepreneurs and education scholars (Fayolle et al., 2016; Jena, 2020; Yuan et al., 2020). Entrepreneurship education at colleges and universities is one of the main sources of improving the individual entrepreneurship quality, entrepreneurship knowledge, and skills (Chusimir, 1988; Galloway and Brown, 2002), and entrepreneurship education at colleges and universities has also been highly recognized by the society (Liu et al., 2019; Jena, 2020; Yuan et al., 2020). From 2013 to 2020, the Government of China released more than 100 policy documents to support and encourage the development of “entrepreneurship education.” In recent years, the number of entrepreneurship courses at colleges and universities has been continuously increased because the government and the colleges and universities expect to cultivate the students’ innovative thinking and entrepreneurial ability through entrepreneurship education so as to promote the employment quality of college graduates, create new employment posts, and finally make contributions to the economic growth. Therefore, exploring the operational principle of entrepreneurship education is the primary task.

Entrepreneurship education has become an important link of talent cultivation at colleges and universities. At present, research on entrepreneurship education focuses on the construction of entrepreneurship education ecosystem (Ruskovaara and Pihkala, 2015; Wei et al., 2019), course development of teaching personnel (Falck et al., 2016), whether entrepreneurship education will affect entrepreneurial intention (Martin et al., 2013; Pittaway and Cope, 2016), and so on. According to the social cognitive theory, individuals will pursue their targets only when they believe their abilities and actions can achieve the expected results (Bandura et al., 2003; Bandura, 2018). However, there are great differences between the degree of entrepreneurship education and students’ entrepreneurial intention at different colleges and universities in different areas. Can entrepreneurship education at universities effectively promote the students’ entrepreneurial intention? In the previous literature, their relationship was researched, but inconsistent conclusions were made. Some researchers believe that the positive role played by entrepreneurship education in entrepreneurial intention cannot be ignored (Lüthje and Franke, 2003; Athayde, 2009; Liu et al., 2019), while other researchers believe that entrepreneurship education does not play a significant role in students’ entrepreneurial intention and even plays a negative role (Graevenitz et al., 2010; Yukongdi and Lopa, 2017). Some scholars also suggest that college students should not understand entrepreneurship through entrepreneurship education because entrepreneurship education “may” be helpless to a successful career. Therefore, the influence of entrepreneurship education on entrepreneurial intention should be further verified.

Entrepreneurship education is aimed at cultivating innovative talents, which are the important driving force for future development (Zhang et al., 2014; Lv et al., 2021; Zhuo et al., 2021). Innovation is regarded as an internal driving force, and innovation is related to the mental state of entrepreneurs. Start-ups are more capable of learning and may promote the enterprise performance through learning, imitation, transformation, and re-innovation (Christensen, 1995; Teece, 2007; Teece and Leih, 2016; He et al., 2019; Yuan and Wu, 2020). According to the innovation and entrepreneurship theory, entrepreneurial strategies mainly include two innovation types: exploration innovation and exploitation innovation (Christensen, 1995; Teece, 2007; Jansen et al., 2009). Exploration innovation is characterized by high risk, uncertainty, and uniqueness, while exploitation innovation is characterized by practicability, efficiency, and learning. Although they are correlated, they are different and competitive (Huang, 2019). Exploration innovation and exploitation innovation need to be supported by innovation resources such as knowledge, technologies, talents, and funds, and enterprises need strong innovation governance ability when searching, finding, selecting, controlling, and utilizing the innovation resources (Huang, 2019). However, there is a lack of discussion on the mediating effect of entrepreneurial strategy on entrepreneurship education and entrepreneurial intention.

Based on the resource-based theory, entrepreneurship needs to integrate the internal and external resources, and local government subsidies are one of the important external resources to support the start-ups (Bacigalupo et al., 2016; Tsai and Huang, 2016; He et al., 2019; Wu et al., 2020). Government subsidies are aimed at promoting the enterprise innovation ability and performance to build national competitiveness. Government subsidies, with the financial incentives, support measures, and protection policies, have been included in the relevant regulations as an innovation policy, generally including tax preference, rent reduction and exemption, research and development subsidies, technology and talent introduction, market development, management consulting, patent and intellectual property protection, to provide support and guarantee for the professional, directional, and forward-looking development of the industry as well as new activities initiated by the enterprises. In the context of “mass entrepreneurship and innovation,” in recent years, local governments have increasingly strengthened the establishment of industry funds, investment in research and development, expenditures, and tax preference. The governments’ innovation policies have encouraged the enterprises’ investment in the product research and development expenditures, resulting in higher return to the society. However, the regulation role of government subsidies in entrepreneurial strategies and entrepreneurial intention lacks the empirical evidence.

In this article, based on the social cognitive theory and resource-based theory, the research model of the influence of entrepreneurship education on entrepreneurial strategy and entrepreneurial intention is built to thoroughly explore and analyze the influence mechanism of entrepreneurship education on entrepreneurial intention and verify the regulating effect of government subsidy policy. In this article, the research and application of cognitive behavior theory in entrepreneurship education are expanded (Baum et al., 2001; Morris et al., 2013; Zhang et al., 2014; Lv et al., 2021) to provide the theoretical basis for the construction of entrepreneurship education ecosystem, which is conductive to innovation and entrepreneurship to promote the regional economic development.

## Literature Review and Hypotheses

### Entrepreneurship Education and Entrepreneurial Intention

Entrepreneurship education first guides the students to confirm their own entrepreneurial intention. Based on the social cognitive theory, individuals tend to pursue their own targets only when they believe their abilities and actions can achieve the expected results (Bandura et al., 2003; Bandura, 2018). Entrepreneurship education is helpful to improve their cognition, continuously adjust their thoughts and actions to make their entrepreneurship more directional, coherent, and meaningful. Entrepreneurship education integrates all entrepreneurship knowledge transmission forms, and entrepreneurial intention is a mental state that promotes the individuals to form the new concept of business (Bird, 1988). The relationship between entrepreneurship education and entrepreneurial intention was researched in previous literature, and a conclusion was drawn: Entrepreneurship education can significantly influence the students’ entrepreneurial intention (Lüthje and Franke, 2003; Izedonmi and Okafor, 2010). Robinson and Haynes (1991) found in the research that entrepreneurship education can promote the students’ perception of feasibility by increasing the students’ knowledge to improve their entrepreneurial intention. Dyer (1994) believed that there is a relation between entrepreneurship education and students’ entrepreneurial intention because entrepreneurship education can cultivate the students’ confidence in entrepreneurship. Martin et al. (2013) found in the research that entrepreneurship education not only could promote the students’ entrepreneurship knowledge and skills but also could significantly promote the students’ entrepreneurial intention.

Whether entrepreneurship could be taught was a hot topic in the academic circle once. There was a viewpoint: Entrepreneurs can only be identified instead of being “produced” (Adcroft et al., 2004). However, there was another viewpoint: Although entrepreneurs’ characteristics are personal, they can be cultivated by unconventional teaching methods (Kirby, 2004). Some scholars indicated that “In modern society, the role of education in cultivating entrepreneurs is underestimated,” because more than 90% of the successful founders of high-tech companies are college graduates, and more than half of them have graduate degrees (Marvel, 2013; Yuan et al., 2021). Wilson et al. (2007) thought that entrepreneurship education can promote the generation of entrepreneurial intention (Zhang et al., 2014; Lv et al., 2021). Therefore, Hypothesis 1 is proposed in this article:

*Hypothesis 1*: Entrepreneurship education and entrepreneurial intention are positively correlated.

### Relationship Between Entrepreneurship Education, Exploration Innovation, and Entrepreneurial Intention

Gorman et al. (1997) thought that entrepreneurship education is used to cultivate the students’ entrepreneurial quality and intention through the entrepreneurship courses. Brown (2000) defined entrepreneurship education as an activity cultivating the students’ entrepreneurship values, entrepreneurial skills, and the ability to seek business opportunities. In the entrepreneurship framework put forward by Bacigalupo et al. (2016), he regarded opportunity identification, entrepreneurial skill, and action as three key indicators of entrepreneurial ability. Riahi (2010) thought cultivating the students’ entrepreneurial spirit and entrepreneurial attitude in entrepreneurship education was more important than training the students’ entrepreneurial thinking and entrepreneurial strategies. Entrepreneurship education focuses on cultivating innovative skills that can be applied to practice and education and can support innovation in the environment (Binks et al., 2006; Lv et al., 2021; Zhuo et al., 2021). Undergraduate entrepreneurship utilizes multiparty interaction to realize the knowledge iteration in the learning network, and innovation process is the result of interaction of environment, organizations, and entrepreneurs (Anderson et al., 2014; Gundry et al., 2014). Entrepreneurial abilities include the adaptive behaviors and strategies influencing others’ behaviors in the relationship environment (Ferris et al., 2005; Tocher et al., 2012; Lv et al., 2021) so as to drive innovation and bring high returns.

Exploration innovation, which is one of the entrepreneurial strategies, makes destructive innovation based on new knowledge, technologies, design, and products to create customer value (Jansen et al., 2009; He et al., 2019). March (1991) thought that exploration innovation and exploitation innovation were very important for enterprises to keep the competitive edge and develop sustainably and that the knowledge required by exploration innovation was novel and special and characterized by searching, finding, high risk, test, and flexibility. The start-ups need a lot of resources and new technologies in the process of exploration innovation. Through exploration innovation, the start-ups may obtain new knowledge, technologies, and processes to adapt to the changes in the market environment, enter the new product or market field, and even bring new niches. However, enterprises tend to focus on exploration innovation, which may bring high risks to obtain immature ideas and cannot establish a unique competitive edge, resulting in the failure of “exploration–failure–re-exploration.” Entrepreneurship education provides multiple channels for undergraduate entrepreneurs to obtain the resources, such as providing entrepreneurship knowledge and skill cultivation (Chusimir, 1988; Galloway and Brown, 2002). The social network of human capitals built by highly skilled undergraduate entrepreneurs has enhanced the ability of entrepreneurship teams to obtain the resources, reduce the cost of obtaining the resources, and promote the entrepreneurs’ intention to share the knowledge. Through mutual benefit, the resource access and the existing resources are integrated to generate new knowledge and make positive contributions to innovation (Tolstoy, 2009).

Entrepreneurs must be engaged in three important tasks, mainly identifying and exploiting opportunities, taking risks, and innovating (Chandler and Hanks, 1994; Fillis and Rentschler, 2010). Identification of entrepreneurial opportunities is a core activity of undergraduate entrepreneurship in the early stage; it is the process of correctly understanding and judging the market demands and continuously dealing with the relevant resources from entrepreneurship learning so as to shape its innovation ability and personality. Such ability is usually developed through the experience of learning (Mitchelmore and Rowley, 2010; Huang et al., 2020), and social learning is an iterative process of learning, action, introspection, and continuous cooperation. The iterative learning process is considered to be a key component to adapt to environmental changes. The shaping process of undergraduate entrepreneurial ability is a social interaction process in which the information resources are obtained and transformed in the forms of observing or directly participating in entrepreneurship education. This process also includes creating new knowledge through transforming experience and applying knowledge to practice (Yukongdi and Lopa, 2017; Huang et al., 2020).

Since entrepreneurship is a dynamic process with uncertainty and high risk, and the start-ups need to be guaranteed with innovation resources in the innovation process, undergraduate entrepreneurship will have high financial costs when it relies on their own resources in the entrepreneurship to implement innovation activities to promote competitive ability so as to achieve the operation goals. Undergraduate entrepreneurship will also face such huge risks as insufficient organization and management ability, large investment in product research and development expenditures, and high cost of improving the processes; therefore, undergraduate entrepreneurship often focuses on the entrepreneurship environment, such as research and development expenditures of the government, tax preference, financial subsidies, and other innovation policies. Therefore, the significant influence of entrepreneurship education on exploration innovation and entrepreneurial intention is researched in this article, and the research Hypothesis 2 and Hypothesis 3 are inferred on this basis:

*Hypothesis 2:* Entrepreneurship education and exploration innovation are positively correlated.
*Hypothesis 3:* Exploration innovation and entrepreneurial intention are positively correlated.

### Relationship Between Entrepreneurship Education, Exploitation Innovation, and Entrepreneurial Intention

Entrepreneurship education is aimed at developing all the basic entrepreneurial skills to achieve successful entrepreneurship (Lazear, 2004; Baron, 2006; Audretsch et al., 2016; Messersmith and Chang, 2017). Traditional entrepreneurship knowledge learning cannot meet the demands of a dynamic environment for entrepreneurial ability. Entrepreneurship education builds a multilevel social network and comprehensive learning management for the professional abilities of entrepreneurs. Entrepreneurship education cultivates the students’ entrepreneurial skills to enable them to cope with the uncertainties and new challenges in the environment (Brian and Norma, 2010; Seikkula-Leino, 2011; Premand et al., 2016; Lv et al., 2021). The entrepreneurship course system lays the foundation for the comprehensive promotion of students’ entrepreneurial ability. From observation to participation, the social learning network provides the student entrepreneurs with multilevel learning channels to continuously improve their learning and practice skills. Therefore, entrepreneurship education can enhance the students’ confidence to solve new and unexpected problems.

Exploitation innovation is another type of entrepreneurial strategy, which is the progressive innovation based on existing technologies, products, and services to meet customer and market demands (Jansen et al., 2009; He et al., 2019). Exploitation innovation meets customers’ demands based on the existing business, and mainly strengthens the existing knowledge, technology, product, and process to improve or expand the products’ efficiency, with the features of production, efficiency, quality, lean, and marketability. March (1991) thought that through comparison, exploitation innovation had a higher success rate and would promote the application by enterprises in the organization, process, product, and service process and would finally cause “the success trap” after generating new knowledge through the “learning–transformation–re-internalization” process. Exploitation innovation tends to construct innovation ability on the existing basis to enhance the enterprise competitive ability and sustainable development, including multidimensional innovation abilities such as construction technology, organization, product, process, and service. Undergraduate entrepreneurs obtain the resources through entrepreneurship education, identify effective knowledge from a large amount of information and process, and integrate them into the new products or services, to seek new niches, increase the success opportunities, and make contributions to the start-ups.

The development of new products and their entry into the new market are the result of entrepreneurial spirit (Miller, 1983; Covin and Slevin, 1989; Wei et al., 2019). The competitive edge of an enterprise may come from efficiency and ability generated by new product development, and R&D investment activities have been considered as the focus of enterprise innovation by many scholars in research. He and Wong (2004) found in the research that enterprises’ exploitation innovation had positive significant influence on the sales growth and enterprise performance. Entrepreneurship education is an important approach for the entrepreneurs to obtain the resources, promote the innovation ability and innovative personality, and integrate various knowledge and value systems to build the multilevel learning channels for the entrepreneurs (Huang et al., 2020; Lv et al., 2021; Zhuo et al., 2021). From knowledge learning to skill promotion, entrepreneurship education includes the development of general abilities and the improvement of professional abilities. Entrepreneurial ability is critical to success, mainly identifying the opportunities and developing necessary resources and capitals (Arthurs and Busenitz, 2006; Kettunen et al., 2013; Liu et al., 2019), as well as technology, financial and legal knowledge (Kuratko, 2005). Considering the diversified entrepreneurial abilities, Bacigalupo et al. (2016) built a framework of entrepreneurial abilities, including opportunity identification, entrepreneurial skills representing “resources,” action fields, and 15 ability lists.

Therefore, the significant influence of entrepreneurship education on exploitation innovation and entrepreneurial intention is researched in this article, and the research Hypothesis 4 and Hypothesis 5 are inferred on this basis:

*Hypothesis 4:* Entrepreneurship education and exploitation innovation are positively correlated.
*Hypothesis 5:* Exploitation innovation and entrepreneurial intention are positively correlated.

### Regulating Effect of Government Subsidies

Government subsidy, which is used by the national or local governments to encourage and support undergraduate entrepreneurship, is an innovation policy to drive economic development and plays the role of stimulation and regulation in regional economic growth and innovation activities. In other words, after the start-ups obtain the government subsidies, they may promote the technical innovation ability by researching and developing new products, introducing new technologies, talents, and equipment as well as organizing and serving innovation, and may also promote the scale benefit through such economic means as expanding the production scale and investment so as to improve the enterprise performance. Exploitation innovation of enterprises will not affect the financial performance but will erode the enterprise resources (Jansen et al., 2009). Since innovation is a dynamic process with uncertainty and high risk, and enterprises need to be guaranteed by innovation resources in the innovation process, enterprises will have high financial costs and will also face such huge risks as the insufficient organization and management ability, large investment in product research and development, and high cost of improving the processes when they rely on their own resources to implement innovation activities to promote competitive ability so as to achieve the operation goals. Therefore, they need the innovation policies of the government, such as investment in research and development, tax preference, and financial subsidies.

According to the resource-based theory, government subsidy is a public policy to play the role of stimulation or encouragement (Bacigalupo et al., 2016; Tsai and Huang, 2016; He et al., 2019), which is characterized by direct subsidy and indirect subsidy. Enterprises obtaining subsidies from the government is a kind of free transfer, which can increase the enterprise cash flow in a short period so as to enhance the competitive edge of the enterprises. The regulating effect of local government subsidy policy on exploration innovation and exploitation innovation, and the research Hypothesis 6 and Hypothesis 7, is inferred on this basis:

*Hypothesis 6:* Government subsidies have a positive regulating effect on exploration innovation and entrepreneurial intention.
*Hypothesis 7:* Government subsidies have a positive regulating effect on the exploitation innovation and entrepreneurial intention.

**FIGURE 1:**
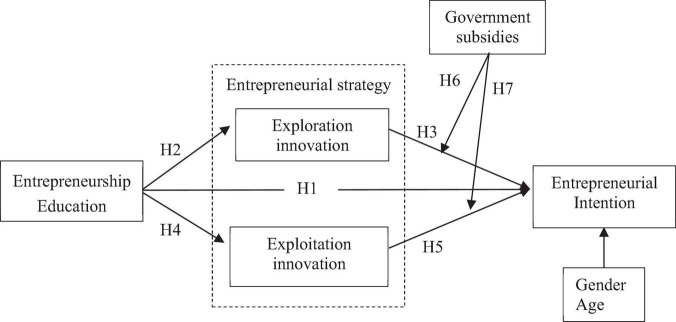
Theoretical conceptual and research framework.

In conclusion, this article is based on the cognitive behavioral theory and the innovation and entrepreneurship theory to analyze the influence of undergraduate entrepreneurship education on entrepreneurial strategies and entrepreneurial intention, and to verify the regulating effect of the government subsidy policy, and its research framework is shown in Figure 1.

## Research Design

### Sample and Data Collection

Innovation resources at home and abroad are gathered in China’s Pearl River Delta to promote mass entrepreneurship and innovation and accelerate the formation of the innovation-oriented economic system and development mode, with the reputation of the capital of innovation. An international first-class innovation and entrepreneurship center is being built. The university encourages undergraduates to participate in innovation and entrepreneurship competitions and provides places to cultivate makers. In this research, with the students at colleges and universities in Pearl River Delta in China as the sampling objects, random samples of the written questionnaire were taken among the students in the education and training classes of *Undergraduate Entrepreneurship* opened by colleges and universities with the support of Human Resources and Social Security Department of Guangdong Province. 1,000 questionnaires were distributed, and 516 questionnaires were collected, with a questionnaire recovery rate of 51.6%. After eliminating 109 questionnaires with incomplete information, there were 334 effective questionnaires, with an effective rate of 64.73%. Among the samples, public comprehensive universities accounted for 31.44%; public application-based universities accounted for 44.31%; independent colleges accounted for 12.57%; and private colleges accounted for 11.68%. Among the majors, science and engineering majors accounted for 33.53%; economy and management majors accounted for 46.11%; humanities/arts majors accounted for 9.88%; medical majors accounted for 1.8%; and agricultural majors accounted for 8.68%; with respect to the degree of education, undergraduate students accounted for 79.64%, and junior college students accounted for 20.36%. Among the questionnaire fillers, men accounted for 60.48% and women accounted for 39.52%. The average age was 24. The results are shown in Table 1.

**TABLE 1:** Distribution of the sampled firms.

	*N* = 334	Percentage
**Type of colleges and universities**		
Public comprehensive university	105	31.44%
Public application-based university	148	44.31%
Independent college	42	12.57%
Private college	39	11.68%
**Major**		
Science and engineering	112	33.53%
Economics and management	154	46.11%
Humanities/arts	33	9.88%
Medicine	6	1.80%
Agriculture	29	8.68%
**Degree of education**		
Undergraduate student	266	79.64%
Junior college student	68	20.36%
**Gender**		
Male	202	60.48%
Female	132	39.52%

### Measures

The development of questions in the questionnaire is mainly referred to the previous theoretical basis and relevant literature. To make the questionnaire rigorous, after the questionnaire was designed, the scholars and professors in relevant fields and the experts of the practice field were invited to provide guidance opinions. After several modifications of questions and grammars, 30 undergraduates from the start-ups were selected for the pre-test, and the formal questionnaires in this study were completed to ensure the accuracy, adaptability, and convenience of answers after the statistical analysis on the pre-test data and after the inappropriate semantics was repeatedly corrected. The questionnaire adopted a 7-point Likert scale. The larger the number, the more you agree with the description (1: strongly disagree; 3: general; 7: strongly agree). The questionnaire construct variables mainly include entrepreneurship education, entrepreneurial strategies of exploration innovation and exploitation innovation, and entrepreneurial intention and government subsidies. The operational definitions of variables and the basis of the research scales in the research framework are described as follows:

#### Entrepreneurship Education

Entrepreneurship education measured in this study focuses on the perspective of social cognition, which is analyzed from three aspects, namely, environment, organization, and personal learning and behaviors. Through the interviews with the full-time teachers, we may understand the main concerns of the entrepreneurship education at colleges and universities, such as entrepreneurship atmosphere, entrepreneurship course, and entrepreneurial activities. In addition, based on the references of Franke and Lüthje (2004), Qi (2017), six programs were proposed to measure the education scale of individual entrepreneurs participating in entrepreneurship education, such as “a creative university campus atmosphere stimulates your entrepreneurial dream,” “entrepreneurship course learning provides the knowledge required by you for the entrepreneurship,” and “universities provide office spaces and entrepreneurship tutors for you.” The Cronbach’s alpha coefficient of this scale is 0.89, which indicates good reliability.

#### Exploration Innovation

Exploration innovation, which is a destructive innovation, is based on new knowledge and technologies to create customer value. Based on the scale of Jansen et al. (2009), its question examples include five questions, such as “destructive innovation” and “develop a new market.” The Cronbach’s alpha coefficient of this scale is 0.87, which indicates good reliability.

#### Exploitation Innovation

Exploitation innovation, which is a progressive innovation, is based on the existing technologies and products to meet customer and market demands. Based on the scale of Jansen et al. (2009), its question examples include four questions, such as “progressive innovation” and “improve the existing product quality and production technologies.” The Cronbach’s alpha coefficient of this scale is 0.91, which indicates good reliability.

#### Entrepreneurial Intention

Entrepreneurial intention is mainly based on the scale proposed by Liñán and Chen (2009), including six questions, such as “I’m yearning for establishing my own enterprise” and “I’m confident that I always have the entrepreneurship enthusiasm to achieve the successful entrepreneurship.” The result indicates that the Cronbach’s alpha coefficient of this scale is 0.86, which has good reliability.

#### Government Subsidies

Government subsidies are mainly based on the scale proposed by Huang (2019), and the constructs contain direct subsidy and indirect subsidy, including nine questions such as providing the interest-free loan, technical funding, research and development subsidies, tax preference, talent reward, and product certification funding, and the question examples are as follows: “Talent introduction reward, training and housing purchase subsidy” and “the government provides the loan without interest or with interest subsidy.” The result indicates that the Cronbach’s α coefficient of this scale is 0.92, which has good reliability.

#### Control Variable

In this study, gender, age, and degree of education are taken as control variables (Brockhaus, 1980) to eliminate their influence on entrepreneurial intention.

## Empirical Analysis

### Average Verification

In this study, SPSS23.0 statistical analysis software is used in descriptive statistical analysis on the sample data, and the mean value, SD, and correlation coefficient of each variable are shown in Table 2. According to Table 2, the correlation coefficients of entrepreneurship education, entrepreneurial strategies of exploration innovation and exploitation innovation, government subsidies, and entrepreneurial intention are between 0.11 and 0.71 and reach a significant level, showing a moderate positive correlation between each factor and entrepreneurial intention. Considering that each questionnaire is filled by the same respondent, and there may be common method variance (CMV) in the data, so Harman’s single-factor method is used to solve the CMV problem. It is found that in the circumstance of no rotation, the first factor explains 32.45% of the variance, which is not more than half. It shows that the common method variance in this research does not affect the results of research (Huang et al., 2020; Wu et al., 2020).

**TABLE 2:** Basic descriptive statistics of the correlation coefficients.

Variable	Mean	Std	1	2	3	4	5	6	7
1. Entrepreneurial intention	4.31	1.52	0.86						
2. Entrepreneurship education	5.44	1.30	0.11*	0.80					
3. Exploration innovation	4.68	1.68	0.53**	0.16**	0.79				
4. Exploitation innovation	4.90	1.26	0.48**	0.19**	0.71**	0.86			
5. Government subsidies	5.37	1.26	0.29**	0.35**	0.57**	0.59***	0.89		
6. Gender	3.24	1.05	0.01	–0.02	–0.11	–0.53	–0.02	1	
7. Age	3.49	0.82	0.13	–0.36	–0.03	–0.10	–0.01	0.53**	1
CR			0.95	0.90	0.89	0.92	0.97		
AVR			0.74	0.64	0.62	0.75	0.79		

The diagonal value represents the square root of the average variance extracted (AVE) of the construct. The off-diagonal value represents the correlation coefficient of each construct. N = 334; *p < 0.05; **p < 0.01; ***p < 0.005 (two-tailed test).

### Confirmatory Factor Analysis

In this study, Mplus7.0 statistical analysis software is used to analyze the reliability, convergent validity, and discriminatory validity of the questionnaire scale with the confirmatory factor. See Table 2 for the results. All Cronbach’ α coefficients of the research variables are greater than 0.7%; their combined reliability value is greater than 0.7, the average variance extraction reaches 0.5, and the convergent validity reaches the standards suggested by the relevant scholars (Fornell and Larcker, 1981; Anderson and Gerbing, 1988). The confidence interval test method is used for discriminatory validity. The test results show that the upper and lower values of the correlation coefficient between the constructs after adding and subtracting two SEs do not contain 1, which meets the standard for a good discriminatory validity (Bagozzi and Yi, 1988). CFA result shows χ^2^ = 1,121.84, df = 382, cmin/df = 2.93 < 3, *p* < 0.001; SRMR = 0.05 < 0.08; CFI = 0.92 > 0.90; TLI = 0.91 > 0.90; RMSEA = 0.06 < 0.08, indicating that the questionnaire has relatively good reliability and validity and that the scale has good measurement quality (Hu and Bentler, 1999; Huang et al., 2020).

### Hypothesis Testing

In this study, the path relationship among various research constructs is discussed through path analysis to verify the research hypotheses proposed in this article. Refer to Table 3 for the summary of the hypothesis testing path coefficient, *t*-value, and results. The model matching degree indicator and standardization path coefficient value (χ^2^ = 1,167.62, df = 471, cmin/df = 2.48, *p* = 0.000; CFI = 0.93; TLI = 0.92; SRMR = 0.05; RMSEA = 0.06) show the good matching degree of the structural models and the observation data.

**TABLE 3:** Hypothesis test and result.

Hypothesis test	Path coefficient	*t*	Result
H1: Entrepreneurship education → entrepreneurial intention	0.03	0.58	Not supported
H2: Entrepreneurship education → exploration innovation.	0.21***	3.55	Supported
H3: Exploration innovation → entrepreneurial intention.	0.34***	4.38	Supported
H4: Entrepreneurship education → exploitation innovation.	0.25***	4.36	Supported
H5: Exploitation innovation → entrepreneurial intention.	0.30***	3.83	Supported
H6: Government subsidies have a positive regulating effect on exploration innovation and entrepreneurial intention.	0.13*	1.46	Supported
H7: Government subsidies have a positive regulating effect on exploitation innovation and entrepreneurial intention.	0.48**	2.96	Supported

N = 334; *p < 0.05; **p < 0.01; and ***p < 0.005.

First, gender and age are taken as control variables to verify the influence of entrepreneurship education, exploration innovation, and exploitation innovation on entrepreneurial intention, and the results (see Table 3) show that entrepreneurship education has no significant influence on entrepreneurial intention (β = 0.03, *t* = 0.58). If Hypothesis 1 is not supported, entrepreneurship education will not directly affect the undergraduate entrepreneurial intention; exploration innovation has the positive significant influence on entrepreneurial intention (β = 0.34^***^, *t* = 4.38). If Hypothesis 3 is supported, exploitation innovation has the positive significant influence on entrepreneurial intention (β = 0.30^***^, *t* = 3.83). If Hypothesis 5 is supported, undergraduate entrepreneurial strategies of exploration innovation and exploitation innovation have a positive influence, and exploitation innovation can better adapt to the market demand, so the entrepreneurship success rate will be higher.

Second, the influence of entrepreneurship education on exploration innovation and exploitation innovation is verified. Entrepreneurship education has a positive significant influence on exploration innovation (β = 0.21^**^, *t* = 3.55), and Hypothesis 2 is supported; entrepreneurship education has a positive significant influence on exploitation innovation (β = 0.25^**^, *t* = 4.36), and Hypothesis 4 is supported, indicating that entrepreneurship education has a positive influence on both exploration innovation and exploitation innovation. Hypothesis 1 is not supported, indicating that entrepreneurship education will not directly affect entrepreneurial intention, but it promotes entrepreneurial intention through the entrepreneurial strategies of exploration innovation and exploitation innovation, so exploration innovation and exploitation innovation have the mediating effect.

**FIGURE 2:**
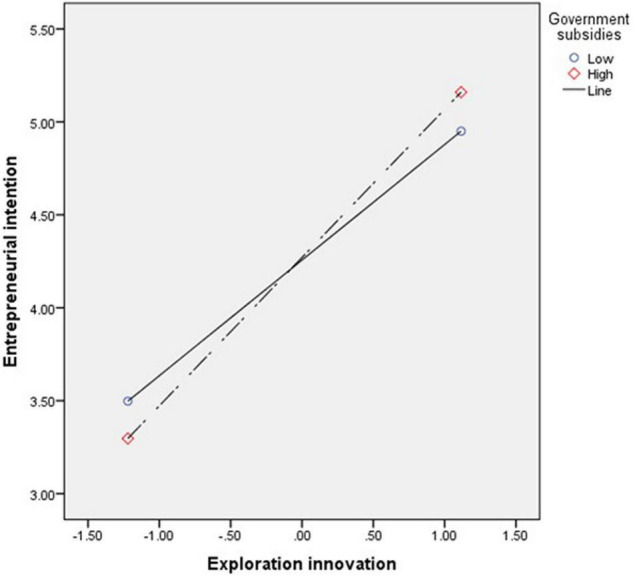
The interaction effect of exploration innovation and government subsidies on entrepreneurial intention.

Finally, the regulating effect of government subsidies is verified. The interaction between government subsidies and exploration innovation has a positive significant influence on entrepreneurial intention (β = 0.13*, *t* = 1.46), and Hypothesis 6 is supported, indicating that government subsidies play a regulating role in exploration innovation at the time of undergraduate entrepreneurship and entrepreneurial intention. As shown in Figure 2, the entrepreneurial environment policy of government subsidies will affect the college students’ entrepreneurial intention. When the government subsidies are high, the college students’ entrepreneurial intention through exploration innovation will be continuously enhanced. When the government subsidies are low, the college students’ entrepreneurial intention through exploration innovation will gradually decline. When the exploration innovation is used in the college students’ entrepreneurial strategy, the market is uncertain and requires a large amount of fund investment, and there is a certain risk in success. Therefore, the local governments shall take the funding measures such as government subsidies and talent incentives to build an entrepreneurial environment conductive to innovation.

**FIGURE 3:**
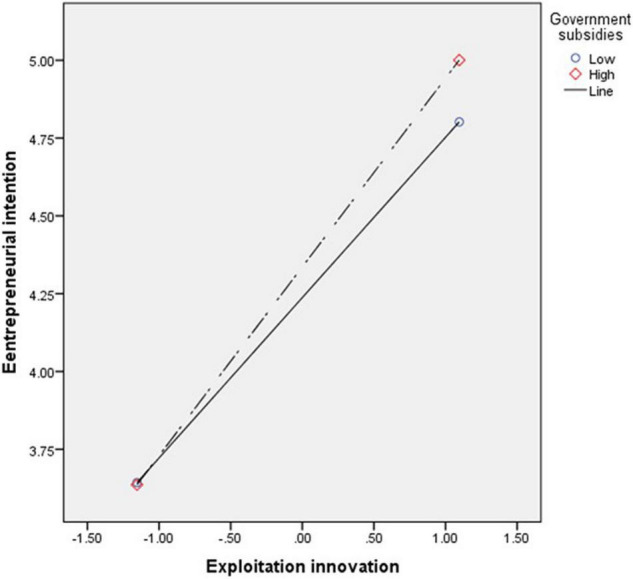
The interaction effect of exploitation innovation and government subsidies on entrepreneurial intention.

The interaction between government subsidies and exploitation innovation has a positive significant influence on entrepreneurial intention (β = 0.48^**^, *t* = 2.96), and Hypothesis 7 is supported, indicating that government subsidies play a regulating role in exploitation innovation at the time of undergraduate entrepreneurship and entrepreneurial intention. As shown in Figure 3, due to the entrepreneurship policy of government subsidies, the college students’ entrepreneurial intention through exploitation innovation is continuously enhanced. The reason is that at the beginning of entrepreneurship, the college students will adopt the entrepreneurial strategy of exploitation innovation to meet the market demands and adapt to the market and survive through simulation learning and that they will invest less while having a great success rate. Therefore, the local governments can drive the college students’ entrepreneurial intention if they provide government subsidies.

## Conclusion

### Conclusion of Research

Based on the social cognitive theory, the mechanism for the influence of entrepreneurship education on entrepreneurial intention is explored and discussed in this article, and the regulating effect of the government subsidy policy is verified. By collecting the data of 334 questionnaires from students at colleges and universities in Guangdong Province, empirical analysis of structural equation is conducted to draw the empirical research conclusions: First, entrepreneurship education has no influence on entrepreneurial intention, which is inconsistent with the conclusion of previous literature research (Robinson and Haynes, 1991; Izedonmi and Okafor, 2010; Wei et al., 2019); that is, entrepreneurship education for students at colleges and universities cannot directly affect the college students’ entrepreneurial intention, the reasons are that the faculty capacity of entrepreneurship education in universities is low, the theory of entrepreneurship education teaching materials is lagging, and entrepreneurship education improves the entrepreneurial intention of college students by improving their entrepreneurial cognitive ability and entrepreneurial skills. Second, entrepreneurship education has the positive significant influence on entrepreneurial strategies of exploration innovation and exploitation innovation; third, entrepreneurial strategies of exploration innovation and exploitation innovation have the positive significant influence on entrepreneurial intention, and exploration innovation and exploitation innovation have a mediating effect on entrepreneurship education and entrepreneurial intention. Finally, government subsidies play a regulating role in exploration innovation and exploitation innovation at the time of undergraduate entrepreneurship and entrepreneurial intention (Messersmith and Chang, 2017; Lv et al., 2021). It can further analyze the influence of entrepreneurship education on entrepreneurial intention so as to expand the research and application of cognitive behavior theory in entrepreneurship education (Baum et al., 2001; Morris et al., 2013; Lv et al., 2021; Zhuo et al., 2021).

### Theoretical Significance

The research results show the influence of entrepreneurship education on entrepreneurial intention as well as the mediating effect of entrepreneurial strategies. Entrepreneurship education not only provides the human capital such as knowledge and skills but also changes the students’ attitudes and behaviors (Liu et al., 2019; Wei et al., 2019). Entrepreneurship education’s influence on the attitude changes as the environment is ignored to a great extent (Medvedeva, 2011; Liu et al., 2019). Social cognitive theory may be used to understand the influence of environmental factors on the individuals’ innovation awareness, innovation ability, and innovation personality. Based on the social cognitive theory, this study postulates that human behaviors are determined by environmental influence, and describes the relationship between having abilities and believing abilities. If individuals believe their abilities and actions can reach the expected result, then they will tend to pursue their own goals (Bandura et al., 2003; Bandura, 2018). Entrepreneurship education shall return to the human essence; cultivate the college students’ characters of “enthusiasm,” “curiosity,” and “persistence”; and guide the students to confirm their willingness to be engaged in entrepreneurship.

The research results verify the regulating effect of the government subsidy policy. Entrepreneurship needs a lot of resources and a lot of funds and has certain risks for success, so the local governments shall take the funding measures such as government subsidies and talent incentives to build an entrepreneurial environment in favor of innovation (Bacigalupo et al., 2016; He et al., 2019). Entrepreneurial ability is multidimensional and dynamic in nature (Zahra et al., 2006; Liu et al., 2019). Focusing on the cultivation of students’ entrepreneurial skills is conducive to achieving the goals of entrepreneurship education organizations and meeting the overall development demands of entrepreneurial activities (Liu et al., 2019; Lv et al., 2021; Zhuo et al., 2021). According to the previous literature, entrepreneurship education is divided into two stages: First, cultivate the spirit of entrepreneurs that all students shall have (Martin et al., 2013; Wei et al., 2019); second, cultivate the students’ entrepreneurial skills such as business mode, marketing strategy, and communication and negotiation (Bacigalupo et al., 2016; Liu et al., 2019). Entrepreneurship is valuable because the individuals may achieve self-worth and may bring development and progress to the state and the society, and the spirit of entrepreneurs and entrepreneurship shall be promoted based on entrepreneurship education. In addition, the spirit of entrepreneurs is an important factor indispensable from the national and social development and progress, and giving play to the spirit of entrepreneurs is the core of economic growth (Fayolle et al., 2016). Therefore, the society shall encourage the young people for entrepreneurship and promote the entrepreneurship education at colleges and universities.

### Management Implication

Improve the entrepreneurship education ecosystem to promote the college students’ entrepreneurial intention. First, establish a perfect entrepreneurship education mechanism. Entrepreneurship education is especially important at the early stage of entrepreneurship career, which enables the students to carefully consider whether to be engaged in entrepreneurship and is conducive to the formation of entrepreneurial intention (Fayolle et al., 2016; Liu et al., 2019; Yuan et al., 2020); second, colleges and universities shall strengthen the construction of undergraduate entrepreneurship education course system, build entrepreneurship incubation bases at colleges and universities, and enrich the students’ entrepreneurship knowledge through courses and competitions to promote their entrepreneurial practical ability and promote the formation of the students’ entrepreneurial intention (Martin et al., 2013; Falck et al., 2016); third, in the context of “mass entrepreneurship and innovation,” the government shall actively provide good policy environment for the college students to be engaged in entrepreneurship and build the “five-in-one” entrepreneurship education ecosystem of government, colleges and universities, enterprises, society, and college students to encourage the college students to be self-employed and promote the development of the national innovative and entrepreneurial economy.

As the subjects of entrepreneurship education learning, the students shall consider their own obvious campus characteristics. Innovation is the power for entrepreneurship program development; in the students’ entrepreneurship, many entrepreneurship programs are based on innovative technology transformation and creativity (Lv et al., 2021; Zhuo et al., 2021). Entrepreneurship education focuses on improving entrepreneurial professional skills based on actions instead of the transfer of theoretical knowledge in class (Kassean et al., 2015). Student entrepreneurs form the learning network in a good entrepreneurship education environment through participating in learning and taking advantage of their own influence to continuously obtain and exchange valuable resources through persuasion and cooperation and building a shared social resource network to promote professional skills. The effectiveness and transformation rate of innovation knowledge enhance the influence of perceptual entrepreneurship education on innovation (Martin et al., 2013; Anderson et al., 2014; Yuan et al., 2020; Lv et al., 2021; Zhuo et al., 2021). In entrepreneurship education, learning the relevant entrepreneurship knowledge is conducive to strengthening the students’ entrepreneurial intention (Lüthje and Franke, 2003; Izedonmi and Okafor, 2010; Liu et al., 2019).

At present, an innovation-driven development strategy puts forward new requirements for entrepreneurship education. As a new incubator of innovative talents, entrepreneurship education shall also focus on promoting entrepreneurial professional ability. Entrepreneurship education at colleges and universities is aimed at “education” and “learning” and inspiring the students’ entrepreneurial dream and interest instead of improving the success probability of entrepreneurship in reality. It is necessary to improve the entrepreneurship education reform in colleges and universities, fully consider the demands and characteristics of undergraduate entrepreneurs, organize various teaching practice activities, and actively participate in the innovation and entrepreneurship competitions.

### Limitation and Future Research Direction

Entrepreneurial intention is affected in multiple aspects and in multiple dimensions. In the future, a new model may be built in the aspects of the identification of entrepreneurial opportunities, the spirit of entrepreneurs, and teaching style. At the same time, the students at colleges and universities as the research object are evaluated for entrepreneurship education. In the future, dynamic tracking may be conducted, and cross-level research of organization and management levels may be conducted.

## Data Availability Statement

The original contributions presented in the study are included in the article/supplementary material, further inquiries can be directed to the corresponding author/s.

## Ethics Statement

This study was carried out in accordance with the recommendations of the Ethics Committee of Macau University of Science and Technology with written informed consent from all subjects in accordance with the Declaration of Helsinki. The protocol was approved by the Ethics Committee of the Macau University of Science and Technology.

## Author Contributions

JL is responsible for the proposal of the research topic, research structure, and manuscript writing. S-ZH is responsible for data analysis, manuscript writing, and revision. KC and LY are responsible for data collection, collation, and analysis, as well as manuscript revision. All authors approved the final version.

## Conflict of Interest

The authors declare that the research was conducted in the absence of any commercial or financial relationships that could be construed as a potential conflict of interest.

## Publisher’s Note

All claims expressed in this article are solely those of the authors and do not necessarily represent those of their affiliated organizations, or those of the publisher, the editors and the reviewers. Any product that may be evaluated in this article, or claim that may be made by its manufacturer, is not guaranteed or endorsed by the publisher.
